# Salty taste test for a low-salt diet to control blood pressure

**DOI:** 10.1186/s40885-023-00236-7

**Published:** 2023-04-15

**Authors:** Seon-Hee Yang, Jea-Chul Ha, Min-Ji Kim

**Affiliations:** 1 Division of Occupational and Environmental Medicine, Dongguk University Gyeongju Hospital, Gyeongju, Republic of Korea; 2 Division of Occupational and Environmental Medicine, Keimyung University Dongsan Hospital, Daegu, Republic of Korea

**Keywords:** Blood pressure, Sodium, Taste sense, Judgment

## Abstract

**Background:**

Reducing salt intake helps prevent complications of cerebrovascular disease. To help patients accept a low-sodium diet, the salty taste test is used to evaluate how much salt an individual actually consumes. The aim of this study was to help patients with hypertension reduce their salt intake by helping them recognize the difference between their subjective perception of saltiness and the objective test results.

**Methods:**

We enrolled workers who visited a local occupational health institution in the period from April to August 2019. Demographic and physical characteristics were recorded. Blood pressure measurement and use of medication were also recorded. A questionnaire was used to investigate whether people liked or disliked salty food, i.e., preference for saltiness, and whether they usually ate salty, normal, or fresh food, i.e., the subjective perception of saltiness. Subsequently, the taste determination kit provided by the Ministry of Food and Drug Safety was used to objectively test saltiness at various salty taste concentrations. The Ministry of Food and Drug Safety program (No. 10-093760) was used as the salty taste judgment tool.

**Results:**

A total of 86 workers were surveyed. Eleven of 18 workers (61.1%) who reported usually eating fresh food actually ate normal or salty food. Thirteen of 37 workers (35.1%) who reported eating normal food actually ate salty food. Thirteen of 31 workers (41.9%) who reported eating salty food actually ate fresh or normal food. Of 46 workers who reported disliking salty food, 14 (30.4%) actually ate salty food, while 20 (43.5%) ate normal food. The subjective perception and preference for saltiness were not significantly correlated with the objective test results (*P* = ​​0.085 and *P* = 0.110, respectively). As for the subjective perception and preference for saltiness, Cohen’s weighted kappa for the taste judgment result were 0.23 and 0.22, respectively, indicating a low degree of agreement.

**Conclusions:**

In dietary counseling to prevent cerebrovascular and cardiovascular diseases, rather than relying on the subjective perception of saltiness, a salty taste test should be performed such that people can recognize their salty food eating habits through objective evaluation.

## Background

Salt intake is associated with hypertension, and increased salt intake increases the risk and mortality of cardiovascular diseases. Although the World Health Organization recommends a daily salt intake < 5 g and sodium intake < 2,000 mg [1], Koreans consume 3,488 mg of sodium, which is 1.7 times higher than the recommended intake [2]. Although the results of domestic and international studies on whether high blood pressure occurs in salt eaters are inconsistent [3, 4], many studies have shown the relationship between salt intake and blood pressure. Reducing salt intake in patients with hypertension positively affects the cardiovascular system, reduces blood pressure, and improves vascular elasticity [1]. Nonpharmacological treatment of hypertension is based on improving dietary habits, such as food intake control, and limiting salt intake is a clinically standardized recommendation [5]. However, dietary compliance is poor [6].

In Korea, where salt is added to all foods except rice and salt intake is high, eating fresh is important to reduce salt intake. However, the frequency of dietary management does not differ in Koreans with and without hypertension, and a standardized approach is necessary to improve blood pressure control in Koreans because patients with hypertension have poor dietary adherence [7]. When an individual reports that they eat fresh or salty, the subjective judgment can be incorrect. Therefore, objectively evaluating whether a person actually eats fresh or salty can help improving an individual’s eating habits.

This study compared the results of the subjective evaluation of the preference and perception for saltiness of workers who visited a local occupational health institution with the results of an objective evaluation through a test. The results of this study could help counsel to adopt a low-sodium diet for cerebrovascular disease management. The aim of this study was to help patients with hypertension reduce their salt intake by helping them recognize the difference between their subjective perception of saltiness and the objective test results.

## Methods

The study was approved by the Research Ethics Committee of Keimyung University (No. DSMC 2021-12-054). From April to August 2019, a salty taste test was conducted for workers visiting a local industrial health institution. Demographic and physical characteristics were recorded. Blood pressure measurement and use of medication were also recorded. The salty taste judgment tool used the Ministry of Food and Drug Safety program (No. 10-093760). First, a questionnaire was used to investigate whether the participants liked or disliked salty food as their preference and whether their usual eating habit includes eating salty, normal, or fresh as per their subjective perception. Subsequently, the salty taste test (strength and preference tests) was performed at five salty taste concentrations using a taste determination kit provided by the Ministry of Food and Drug Safety to objectively determine the degree of saltiness. The saltiness concentration test was conducted using bean sprout soup at five concentrations (0.08%, 0.16%, 0.31%, 0.63%, and 1.25%). The subjective intensity of saltiness was fresh, slightly fresh, moderate, slightly salty, or salty, and the subjective preference for saltiness was dislike, slightly dislike, average, slightly good, or good. The Ministry of Food and Drug Safety’s salty taste test program combines the intensity and preference results at each concentration. Therefore, it was used to evaluate the workers’ subjective perception of saltiness as fresh, somewhat fresh, normal, somewhat salty, or salty. For statistical analyses, the workers were divided into three groups: eat fresh, normal, and salty groups. Relevance was checked with a chi-square test, and concordance was obtained by calculating Cohen’s weighted kappa. Statistical analyses were performed using SPSS ver. 18 (SPSS Inc., Chicago, IL, USA).

## Results

From April to August 2019, the salty taste test was conducted randomly on 86 workers using the five salty taste levels. Table 1 shows the general characteristics of the 86 workers.

**Table 1:** General and occupational characteristics of study participants (*n* = 86)

Characteristics	Salty taste test result			*P*-value
	Fresh (*n* = 20)	Normal (*n* = 31)	Salty (*n* = 35)	
Sex				0.871
Male	4 (20.0)	8 (25.8)	9 (25.7)	
Female	16 (80.0)	23 (74.2)	26 (74.3)	
Age (yr)				0.854
< 50	4 (20.0)	8 (25.8)	6 (17.1)	
50–59	9 (45.0)	13 (41.9)	19 (54.3)	
≥ 60	7 (35.0)	10 (32.3)	10 (28.6)	
Hypertension				0.978
No	14 (70.0)	22 (71.0)	24 (68.6)	
Yes	6 (30.0)	9 (29.0)	11 (31.4)	
Body mass index (kg/m^2^)				0.475
< 23	8 (40.0)	18 (58.1)	14 (40.0)	
23.0–24.9	6 (30.0)	7 (22.6)	8 (22.9)	
≥ 25	6 (30.0)	6 (19.4)	13 (37.1)	
Blood pressure				0.997
Normal	16 (80.0)	25 (80.6)	28 (80.0)	
High	4 (20.0)	6 (19.4)	7 (20.0)	
Antihypertensive drug use				0.620
No	17 (85.0)	25 (80.6)	26 (74.3)	
Yes	3 (15.0)	6 (19.4)	9 (25.7)	
Type of industry				0.290
Manufacturing	7 (35.0)	16 (51.6)	10 (28.6)	
Service	12 (60.0)	13 (41.9)	20 (57.1)	
Other	1 (5.0)	2 (6.5)	5 (14.3)	
Employment status				0.824
Full-time	14 (70.0)	20 (64.5)	25 (71.4)	
Temporary	6 (30.0)	11 (35.5)	10 (28.6)	
Shiftwork				0.305
No	20 (100)	28 (90.3)	31 (88.6)	
Yes	0	3 (9.7)	4 (11.4)	
Company size (no. of workers)				0.137
< 30	11 (55.0)	14 (45.2)	22 (62.9)	
30–99	6 (30.0)	12 (38.7)	4 (11.4)	
≥ 100	3 (15.0)	5 (16.1)	9 (25.7)	

Data are presented as number (%). Statistical analyses were performed using the chi-square test

The participants included 65 women (75.6%) and 21 men (24.6%). Among the participants, 68 (79.1%) were aged over 50 years. Hypertension was diagnosed in 26 participants (30.2%). The body mass index was < 23 kg/m^2^ in 40 workers (46.5%). At the time of the study, 69 workers (80.2%) had a blood pressure < 140/90 mmHg (normal), and 18 workers (20.9%) were on antihypertensive drugs. The service industry employed 45 workers (52.3%), with the employment type being full-time in 59 workers (68.6%) and shift in seven workers (8.1%). Small workplaces with < 30 employees were the most prevalent, with 47 workers (54.7%). The taste judgment did not differ by sex, age, hypertension, body mass index, current blood pressure, current antihypertensive medication use, industry, employment type, or business size (Table 1).

With the objective salty taste test, the eat salty, normal, and fresh groups comprised 35 (40.7%), 31 (36.0%), and 20 workers (23.3%), respectively. In the subjective evaluation of the intensity of saltiness, 37 (43.0%), 31 (36.0%), and 18 workers (20.9%) reported usually eating salty, normal, and fresh, respectively. Regarding the preference for saltiness, 40 (46.5%) and 46 workers (53.5%) answered liking and disliking salty food, respectively.

Of the 18 workers who reported usually eating fresh food, only seven (38.9%) actually ate fresh food, whereas 11 (61.1%) ate normal or salty food. Of the 37 workers who reported usually eating normal food, 16 (43.2%) actually ate normal food, while 13 (35.1%) ate salty food. Of the 31 workers who reported eating salty food, 18 (58.1%) actually ate salty, while five (16.1%) ate fresh food and eight (25.8%) ate normal food.

In the preference evaluation, of the 40 workers who reported liking salty food, 21 (52.5%) actually ate salty food, while 19 (47.5%) ate normal or fresh food. Of the 46 workers who reported disliking salty food, 14 (30.4%) actually ate salty food, while 20 (43.5%) ate normal food and 12 (26.1%) ate fresh food (Table 2).

**Table 2:** Objective evaluation results of the subjective judgment of saltiness (*n* = 86)

Variable	Test result			*P*-value	Weighted kappa
	Fresh	Normal	Salty		
Subjective perception				0.085	0.23
Eat fresh (*n* = 18)	7 (38.9)	7 (38.9)	4 (22.2)		
Eat normal (*n* = 37)	8 (21.6)	16 (43.2)	13 (35.1)		
Eat salty (*n* = 31)	5 (16.1)	8 (25.8)	18 (58.1)		
Preference to salty taste				0.110	0.22
Like (*n* = 40)	8 (20.0)	11 (27.5)	21 (52.5)		
Dislike (*n* = 46)	12 (26.1)	20 (43.5)	14 (30.4)		

Data are presented as number (%). Statistical analyses were performed using the chi-square test

The subjective perception and preference for saltiness were not correlated with the objective test results (*P* = ​​0.085 and *P* = 0.110, respectively). Cohen’s weighted kappa values ​​of the subjective evaluation and preference for saltiness were 0.23 and 0.22, respectively, indicating a low degree of agreement.

## Discussion

For a long time, cerebrovascular and cardiovascular diseases were the second and third leading causes of death in Koreans, respectively. A low-salt diet can reduce blood pressure and the risk of complications in patients with hypertension. In a recent meta-analysis, reducing salt intake showed a dose-response relationship with blood pressure and lowered it, with a greater effect in patients with hypertension and in the nonwhite population [8]. Another study reported a fall in blood pressure of 7.1/3.9 mmHg in patients with hypertension and 3.6/1.7 mmHg in normotensive participants per 100 mmol reduction in urinary sodium [9]. Currently, 92.1% of Korean adults consume excess sodium [10]. Therefore, reducing salt intake should be included in lifestyle improvement. Salty food eating habits should be improved. Particularly, most Korean food items contain salt, except rice; therefore, eating fresh food would reduce salt intake. However, people’s subjective perception or preference for saltiness may be objectively incorrect. In a study from Japan, the preference for saltiness was found to be correlated with daily salt intake, but people who disliked salty food showed excessive salt intake [11]. In this study comparing subjective daily eating habits by saltiness with objective evaluation with a taste test kit for workers, the subjective preference for saltiness was not correlated with the objective test results. Further, the subjective perception of eating salty or fresh food did not correlate with the objective test results. The degree of agreement was also low. The test revealed that 57.3% of workers who reported eating fresh or normal food actually ate salty food, while 30.4% of those who reported disliking salty food usually ate salty food. Thus, people’s subjective perception and preference for saltiness may be objectively incorrect. Therefore, when counseling individuals for a low-salt diet to manage high blood pressure and prevent cerebrovascular diseases, their eating habits should be evaluated using an objective tool.

In this study involving healthy workers, the perception of saltiness did not differ by sex, age, or hypertension. Limitations of the study were that most participants were over 50 years of age, possibly carrying diseases that affect the senses, such as diabetes and that the use of antihypertensive drugs was not considered.

In the future, studies involving patients with diabetes, hypertension, or using antihypertensive drugs should be performed. The relationship between subjective judgment and objective test results should be considered in patients with hypertension or using antihypertensive drugs, and whether or not eating habits improved after recognizing the objective taste perception through a salty taste test should be investigated.

## Conclusions

The results of this study showed no significant relationship between the subjective judgment of saltiness and objective salty taste test results. Therefore, people should objectively recognize their salty taste perception and preference through the salty taste test to self-manage and improve their eating habits for the prevention and management of cerebrovascular diseases. Therefore, rather than relying on subjective evaluations in dietary counseling to prevent cerebrovascular and cardiovascular diseases, an objective evaluation should be performed, and salty taste tests should be utilized to recognize salty eating habits.

## Data Availability

Not applicable.
